# A reassessment of *Anthurium* species with palmately divided leaves, and a reinterpretation of *Anthurium* section *Dactylophyllium* (Araceae)

**DOI:** 10.3897/phytokeys.23.4754

**Published:** 2013-06-10

**Authors:** Thomas B. Croat, Mónica M. Carlsen

**Affiliations:** 1 Missouri Botanical Garden, P.O. Box 299, St. Louis, MO 63166, USA; 2 Department of Biology, University of Missouri–St. Louis, One University Boulevard, St. Louis, MO 63121, USA

**Keywords:** *Anthurium*, molecular phylogeny, palmately divided leaves, palmatisect leaves, palmatifid leaves, section *Schizoplacium*, sectional classification

## Abstract

A reappraisal is made of the *Anthurium* Schott species with palmately divided leaves with 3 or more segments free to the base (i.e. palmatisect leaves), previously recognized as section *Dactylophyllium* Schott (Engler), as well as those species with 5 or more segments united at the base (i.e. palmatifid leaves), formerly placed in section *Schizoplacium* Schott (Engler). New molecular data indicates that several species (*Anthurium pedatum* (Kunth) Schott, *Anthurium pedatoradiatum* Schott, and possibly, *Anthurium podophyllum* (Schltdl. & Cham.) Kunth) should be excluded from section *Schizoplacium*, and other species previously placed in that section cannot be separated from section *Dactylophyllium*. Thus, *Anthurium* section *Schizoplacium* is here synonymized within section *Dactylophyllium* and type species are designated for both groups. This paper also provides an updated description of section *Dactylophyllium* as here emended, listing the 24 accepted taxa now included (20 species and 4 varieties or subspecies), along with their geographic distributions.

## Introduction

**Figure 1.**
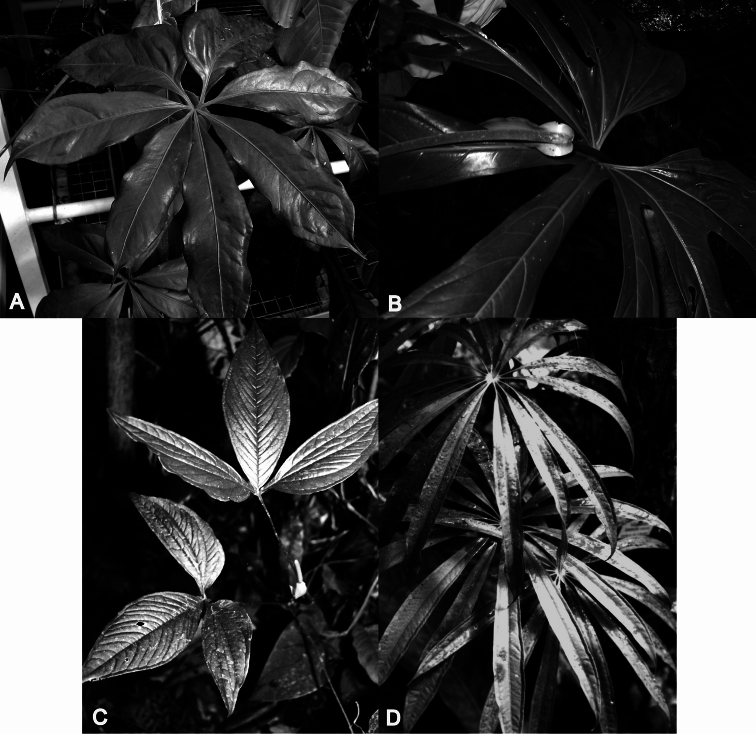
Examples of *Anthurium* species with palmately divided leaves here included in section *Dactylophyllium* (Schott) Engler emend. Croat & Carlsen. **A** Palmatisect leaf with seven leaflets of *Anthurium pentaphyllum* (Aubl.) G.Don var. *pentaphyllum* (*M. Leppard 1395*) **B** Palmatifid leaf of *Anthurium longissimum* Pittier ssp. *longissimum* (*M. Carlsen 2126*) **C** Palmatisect leaves with three leaflets of *Anthurium trisectum* Sodiro (*T.B. Croat 48977*) **D** Palmatisect leaves with more than 9 leaflets of *Anthurium polydactylum* Madison (*T.C. Plowman & H. Kennedy 5769*).

**Figure 2.**
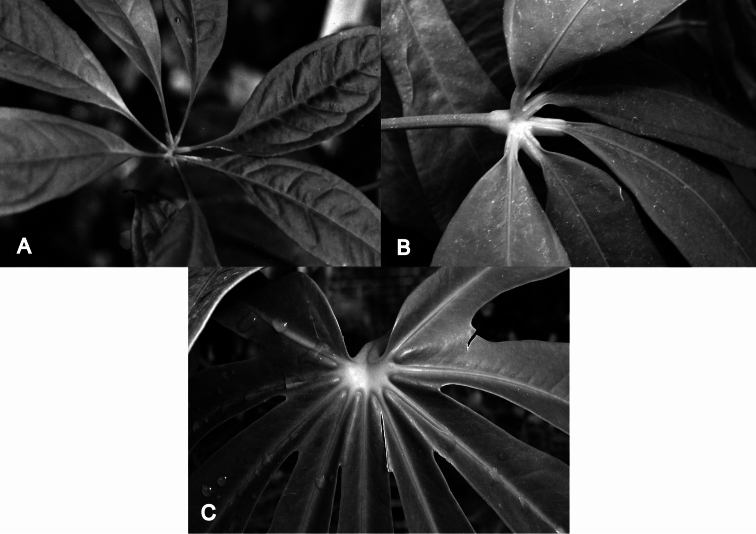
Detail of leaf bases of *Anthurium* species with palmately divided leaves. **A** Palmatisect leaf with segments (leaflets) free to the base with long petiolules, *Anthurium kunthii* Poepp. var. *kunthii* (*J.P. Folsom 3228*) **B** Palmatisect leaf with segments (leaflets) free to the base with short petiolules, *Anthurium pentaphyllum* (Aubl.) G.Don var. *pentaphyllum* (*R.M. Harley 18334*) **C** Palmatifid leaf with segments (lobes) united at the base, *Anthurium palmatum* (L.) Schott (Kew living collection 1980-554).

**Figure 3.**
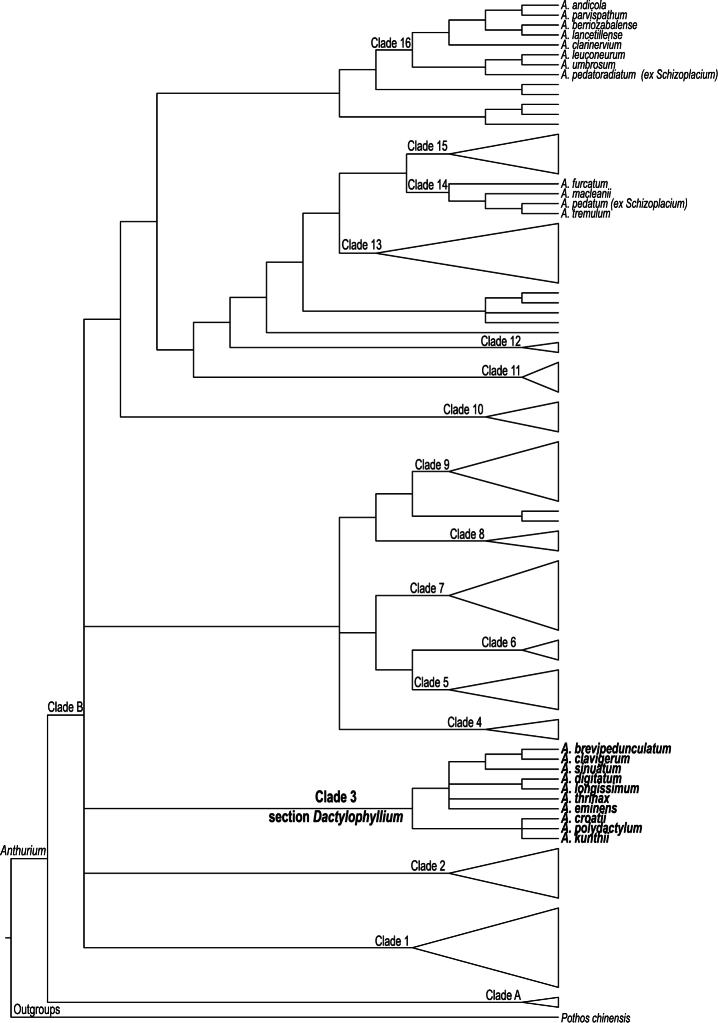
A schematic molecular phylogeny of *Anthurium* showing major clades recovered by Carlsen and Croat (in press). This phylogeny was based on combined chloroplast (*trnG* intron, *trnH–psbA* and *trnC–ycf6* intergenic spacers) and nuclear (first intron of *CHS* and partial flanking coding regions) DNA sequences. Clade numbering follow these authors. Species in bold are recognized here as members of *Anthurium* section *Dactylophyllium* (Schott) Engler, emend. Croat & Carlsen. Placement of *Anthurium* species now excluded from grex *Schizoplacium* Schott is also shown.

*Anthurium* Schott species with palmately divided leaves (as included in Madison 1978) represent a very distinct morphological group within the genus (Fig. 1). In these species, leaf segments (i.e. leaflets) are free to the base, in palmatisect leaves, or leaf segments (i.e. lobes) are united at the base, in palmatifid leaves (Fig. 2). The current sectional classification of *Anthurium* (Croat and Sheffer 1983) separates these species into two groups, section *Dactylophyllium* (Schott) Engler (Engler 1879), comprising species with three or more segments (leaflets) free to the base (Fig. 2A–B), and section *Schizoplacium* (Schott) Engler (Engler 1879), including species with five or more segments (lobes) united at the base (Fig. 2C). A recent molecular phylogeny (Carlsen 2011, Carlsen and Croat in press) has shown that most of the species of *Anthurium* with palmately divided leaves belong to a single highly supported clade (Fig. 3, Clade 3), therefore suggesting that previous divisions of the group are unnecessary. Indeed, the newly circumscribed Clade 3 merits sectional rank. Moreover, although all members of Clade 3 share palmately divided leaves, this leaf form has evolved independently at least two more times within *Anthurium*, in Clades 14 and 16 (Fig. 3). The goal of this study is to reevaluate the limits of sections *Dactylophyllium* and *Schizoplacium* in the light of the new molecular evidence and provide an updated description of this redefined group of *Anthurium* species with palmately divided leaves (Fig. 1).

## Taxonomic history

In the first comprehensive revision of the genus, Schott (1860) classified *Anthurium* species with lobed or divided leaves in three groups (Table 1): grex *Semaeophyllium*, comprising species with “hastate-trilobed” blades with segments united at the base; grex *Schizoplacium*, including species with “pedately-partite” blades with five or more leaf segments united at the base (i.e. palmatifid leaves, according to our definition) (Fig. 2C); and grex *Dactylophyllium*, containing species with “digitisect” leaf blades with three or more segments divided completely (i.e. free) to the base (i.e. palmatisect leaves, in our definition) (Fig. 2A–B).

Carlsen and Croat (2007) recently revised the 23 species included in *Anthurium* section *Semaeophyllium* (Schott) Engler (Engler 1879). The section comprises species with trilobed leaf blades, where leaf lobes are always united at the base, and the lobes can be directed forward (i.e. falcate) or to the sides (i.e. spreading) but never toward the back. On the basis of molecular evidence (Carlsen 2011, Carlsen and Croat in press), section *Semaeophyllium* appears not to be monophyletic. However, species with trilobed leaves are more closely related to other *Anthurium* species with cordate leaves than to the species with palmately divided leaf morphology clustered in Clade 3 (Carlsen 2011, Carlsen and Croat in press). Therefore, this paper will only deal with the *Anthurium* species with palmately divided leaves (Fig. 1), those included in sections *Dactylophyllium* and *Schizoplacium*.

**Table 1.** *Anthurium* species with palmately divided leaves formerly included in *Dactylophyllium* and *Schizoplacium*, a comparison of previous circumscriptions. This is not an exhaustive list of all species names that have been previously included in these groups, it only contains taxa that were accepted at the time of publication of each work. Names in bold denote species included in the newly redefined section *Dactylophyllium* (Schott) Engler emend. Croat & Carlsen, as proposed here. Species marked with (*) are now formally excluded from this emended section. All other species names are either synonyms or species dubia, fide Madison (1978).<br/>

**Species name**	**Year published**	****Schott (1860)****	****Engler (1905)****	****Madison (1978)****	**Croat & Sheffer (1983)**
*Anthurium aemulum* Schott	1859	Dactylophyllium	Schizoplacium series Dactylophyllium	synonym of<br/> *Anthurium pentaphyllum* var. *bombacifolium*	
*Anthurium andersonii* Schott	1857	Dactylophyllium	Schizoplacium series Dactylophyllium	synonym of<br/> *Anthurium palmatum*	
(*) *Anthurium angustisectum* Engl.	1898			Group 6	Schizoplacium
*Anthurium araliaefolium* Regel	1869		Schizoplacium series Euschizoplacium	species dubium, probably a hybrid	
***Anthurium arisaemoides* Madison**	1978			Group 7<br/> Schizoplacium	Dactylophyllium
*Anthurium aubletii* Kunth	1841	Dactylophyllium	synonym of<br/> *Anthurium pentaphyllum*	synonym of<br/> *Anthurium pentaphyllum* var. *pentaphyllum*	
*Anthurium bombacifolium* Schott	1858	Dactylophyllium	synonym of<br/> *Anthurium aemulum*	*Anthurium pentaphyllum* var. *bombacifolium*	
***Anthurium brevipedunculatum* Madison**	1978			Group 7<br/> Schizoplacium	Dactylophyllium
***Anthurium buchtienii* K. Krause**	1910			Group 7<br/> Schizoplacium	Dactylophyllium
***Anthurium clavigerum* Poepp.**	1845	Dactylophyllium	Semaeophyllium	Group 7<br/> Schizoplacium	Dactylophyllium
*Anthurium clavigerum* var. *subpedatipartitum* Engl.	1905		Semaeophyllium	not mentioned	
***Anthurium croatii* Madison**	1978			Group 7<br/> Schizoplacium	Dactylophyllium
***Anthurium digitatum* (Jacq) Schott**	1829	Dactylophyllium	Schizoplacium series Dactylophyllium	*Anthurium pentaphyllum* var. *digitatum*<br/> Group 7<br/> Schizoplacium	not mentioned
*Anthurium elegans* Engl.	1881		Schizoplacium series Euschizoplacium	synonym of<br/> *Anthurium palmatum*	
***Anthurium eminens* Schott**	1855	Dactylophyllium	Schizoplacium series Dactylophyllium	Group 7<br/> Schizoplacium	Dactylophyllium
***Anthurium expansum* Gleason**	1929			Group 6	Schizoplacium
*Anthurium fissum* K. Koch	1864		Semaeophyllium	synonym of<br/> *Anthurium palmatum*	
*Anthurium ghiesbrechtii* Linden ex Schott	1860	Schizoplacium	synonym of<br/> *Anthurium podophyllum*	not mentioned	
*Anthurium grossum* Schott	1859	Dactylophyllium	*Anthurium pentaphyllum* var. *grossum*	synonym of<br/> *Anthurium pentaphyllum* var. *pentaphyllum*	
*Anthurium helleborifolium* Schott	1862		Schizoplacium series Euschizoplacium	synonym of<br/> *Anthurium pedatoradiatum*	
*Anthurium hoffmannseggii* Schott	1857	Dactylophyllium	synonym of<br/> *Anthurium pentaphyllum*	synonym of<br/> *Anthurium kunthii*	
*Anthurium holtonianum* Schott	1857	Dactylophyllium	Semaeophyllium	synonym of<br/> *Anthurium clavigerum*	
*Anthurium holtonianum* var. *cohaerens* Engl.	1905		Semaeophyllium	not mentioned	
*Anthurium kalbreyeri* Mast.	1881		Schizoplacium series Dactylophyllium	synonym of<br/> *Anthurium clavigerum*	
*Anthurium karwinskii* Schott	1859	Dactylophyllium	synonym of<br/> *Anthurium aemulum*	synonym of<br/> *Anthurium pentaphyllum* var. *bombacifolium*	
***Anthurium kunthii* Poepp.**	1845	Dactylophyllium	Schizoplacium series Dactylophyllium	Group 7<br/> Schizoplacium	Dactylophyllium
***Anthurium longissimum* Pittier**	1947			Group 6	Schizoplacium
*Anthurium martini* Schott	1857	Dactylophyllium	Semaeophyllium	synonym of<br/> *Anthurium sinuatum*	
*Anthurium ottonianum* Kunth	1841	Dactylophyllium	*Anthurium variabile* var. *ottonianum*	not mentioned	
*Anthurium pachirifolium* Schott	1855	Dactylophyllium	Schizoplacium series Dactylophyllium	synonym of<br/> *Anthurium pentaphyllum* var. *pentaphyllum*	
*Anthurium pachirifolium* var. *angustifolium* Engl.	1881		Schizoplacium series Dactylophyllium	synonym of<br/> *Anthurium pentaphyllum* var. *pentaphyllum*	
***Anthurium palmatum* (L.) Schott**	1829	Schizoplacium	Semaeophyllium	Group 6	Schizoplacium
*Anthurium panduratum* Mart. ex Schott	1855	Dactylophyllium	Semaeophyllium	synonym of<br/> *Anthurium clavigerum*	
*Anthurium panduratum* var. *burchellianum* Engl.	1905		Semaeophyllium	synonym of<br/> *Anthurium clavigerum*	
(*) *Anthurium pedatoradiatum* Schott	1859	Schizoplacium	Schizoplacium series Euschizoplacium	Group 4	Schizoplacium
(*) *Anthurium pedatum* (Kunth) Schott	1829	Schizoplacium	Schizoplacium series Euschizoplacium	Group 5	Schizoplacium
***Anthurium pentaphyllum* (Aubl.) G. Don**	1839	Dactylophyllium	Schizoplacium series Dactylophyllium	Group 7<br/> Schizoplacium	Dactylophyllium
***Anthurium pentaphyllum* var. *bombacifolium* (Schott) Madison**	1978			Group 7<br/> Schizoplacium	Dactylophyllium
(*) *Anthurium podophyllum* (Schltdl. & Cham.) Kunth	1841	Schizoplacium	Schizoplacium series Euschizoplacium	Group 4	Schizoplacium
***Anthurium polydactylum* Madison**	1978			Group 7<br/> Schizoplacium	Dactylophyllium
***Anthurium polyschistum* R.E. Schultes & Idrobo**	1959			Group 7<br/> Schizoplacium	Dactylophyllium
*Anthurium polytomum* Schott	1859	Schizoplacium	synonym of<br/> *Anthurium podophyllum*	synonym of<br/> *Anthurium podophyllum*	
*Anthurium pseudopodophyllum* Schott	1859	Schizoplacium	synonym of<br/> *Anthurium podophyllum*	synonym of<br/> *Anthurium podophyllum*	
*Anthurium repandum* Schott	1857	Dactylophyllium	Semaeophyllium	synonym of<br/> *Anthurium clavigerum*	
***Anthurium sinuatum* Benth ex Schott**	1857	Dactylophyllium	Semaeophyllium	Group 7<br/> Schizoplacium	not mentioned
*Anthurium smilaciforme* K. Koch	1855	Dactylophyllium	synonym of<br/> *Anthurium undatum*	not mentioned	
*Anthurium sonderianum* Schott	1858	Dactylophyllium	synonym of<br/> *Anthurium pentaphyllum*	synonym of<br/> *Anthurium pentaphyllum* var. *pentaphyllum*	
*Anthurium sylvestre* S. Moore	1895		Semaeophyllium	synonym of<br/> *Anthurium sinuatum*	
***Anthurium thrinax* Madison**	1978			Group 7<br/> Schizoplacium	Dactylophyllium
***Anthurium triphyllum* (Willd. ex Schult.) Brongn. ex Schott**	1860	Dactylophyllium	Schizoplacium series Dactylophyllium	Group 7<br/> Schizoplacium	Dactylophyllium
***Anthurium trisectum* Sodiro**	1905			Group 7<br/> Schizoplacium	Dactylophyllium
*Anthurium undatum* Schott	1832	Dactylophyllium	Schizoplacium series Dactylophyllium	synonym of<br/> *Anthurium pentaphyllum* var. *pentaphyllum*	
*Anthurium undatum* var. *undulifolium* (K. Koch ex Ender) Engl.	1878		Schizoplacium series Dactylophyllium	not mentioned	
*Anthurium undulatum* K. Koch & C. D. Bouché	1854	Dactylophyllium	synonym of<br/> *Anthurium undatum* var. *undulifolium*	not mentioned	
*Anthurium variabile* Kunth	1841	Dactylophyllium	Schizoplacium series Dactylophyllium	synonym of<br/> *Anthurium pentaphyllum* var. *pentaphyllum*	
*Anthurium warscewiczii* K. Koch	1855	Dactylophyllium	synonym of<br/> *Anthurium sinuatum*	not mentioned	
*Anthurium wendlandii* Schott	1858	Dactylophyllium	Semaeophyllium	synonym of<br/> *Anthurium clavigerum*	

Schott (1860) included 27 names in his grex # 28 (Table 1), *Dactylophyllium*, but according to the most updated species synonymy for the genus (Govaerts et al. 2012), only seven species are currently recognized: *Anthurium clavigerum* Poepp., *Anthurium digitatum* (Jacq.) Schott, *Anthurium eminens* Schott, *Anthurium kunthii* Poepp., *Anthurium pentaphyllum* (Aubl.) G.Don, *Anthurium sinuatum* Benth, and *Anthurium triphyllum* (Willd. ex Schult.) Brongn. ex Schott. On the other hand, Schott (1860) included seven names in his grex # 27 (Table 1), *Schizoplacium*, but only four species are now recognized, *Anthurium palmatum* (L.) Schott, *Anthurium pedatoradiatum* Schott, *Anthurium pedatum* (Kunth) Schott, and *Anthurium podophyllum* (Schltdl. & Cham.) Kunth. Engler (1879) gave formal sectional ranking to these, and others, of Schott’s greges, maintaining the species circumscriptions in both groups.

However, Engler (1905) made major modifications in the classification of Schott. He described his newly circumscribed section *Semaeophyllium* as comprising species with “hastate-trilobed or pedatisect or digitisect” leaf blades, and very long and relatively thin (i.e. myosuroideous) spadices. Engler (1905) included in his new version of section *Semaeophyllium*, along with more typical species with trilobed leaves, a pair of species from Schott’s grex *Dactylophyllium* (namely *Anthurium sinuatum* and *Anthurium clavigerum*) and also *Anthurium palmatum*, previously placed by Schott in grex *Schizoplacium*. Alternatively, Engler’s amended section *Schizoplacium* (Engler 1905) included the remaining species of both Schott’s greges *Dactylophyllium* and *Schizoplacium*, along with a few more recently described species, for a total of 17 species, of which only eight are currently accepted (Table 1). Engler’s (1905) new delimitation of section *Schizoplacium* included species with “pedately-partite” leaf blades, with segments either united at the base or completely separated, and thick, conic spadices. He further divided this section into two informal groups, § 1. *Euschizoplacium* Engler, with short stems and internodes, but long peduncles, and § 2. *Dactylophyllium* (Schott) Engler, with scandent stems, elongated internodes, but peduncles often short (Engler 1905). Engler placed most of the species from Schott’s grex *Schizoplacium* in the *Euschizoplacium* group and the remaining species from Schott’s grex *Dactylophyllium* in the *Dactylophyllium* group (Table 1).

The last taxonomic revision of *Anthurium* species with palmately divided leaves (Madison 1978) recognized 27 species and three varieties divided into seven “natural” groupings based on the author’s understanding of the taxonomy, morphology and growth habit of the species (Table 1). Groups 1–3 included species with trilobed leaves with falcate lobes united at the base now placed in section *Semaeophyllium* (Carlsen and Croat 2007). The remaining groups in Madison’s (1978) revision included typical examples of species in sections *Dactylophyllium* and *Schizoplacium* (Table 1; following Croat and Sheffer 1983). Group 4 contained two terrestrial Mexican species with short stems, elongated peduncles and “pedately divided” (i.e. palmatifid) leaf blades (*Anthurium pedatoradiatum* and *Anthurium podophyllum*). Group 5 consisted only of the Colombian species *Anthurium pedatum*, with deeply dissected leaf blades with 11–15 lobes, and a pendent inflorescence borne on an erect peduncle. Group 6 included climbers with palmately divided leaves with the lobes united at the base (i.e. palmatifid leaves) (Fig. 2C), and elongated spadices, which range from northern Colombia to the West Indies (*Anthurium angustisectum* Engl., *Anthurium expansum* Gleason, *Anthurium longissimum* Pittier and *Anthurium palmatum*). The species in Madison’s groups 4, 5 and 6 were placed in section *Schizoplacium* by Croat and Sheffer (1983). His group 7 is a predominantly Amazonian group of species with “digitisect” (i.e. palmatisect) leaf blades, where the leaf segments are free to the base and have a basal pulvinus (Fig. 2A–B), and spadices are purple to gray. Madison called this group section *Schizoplacium*, apparently following Engler’s (1905) circumscription of that section, but it indeed includes species placed in section *Dactylophyllium* by both Schott (1860) and Croat and Sheffer (1983) (Table 1).

Croat and Sheffer (1983) provided the previously accepted treatment of the sections of *Anthurium* with palmately divided leaf blades. Following Schott’s (1860) original classification system, they separated the species of *Anthurium* with lobed or divided leaf blades into three sections, *Semaeophyllium*, *Schizoplacium* and *Dactylophyllium* (Table 1). They provided a key to the sections, descriptions, and illustrative examples of species belonging to each group.

## Results and discussion

The current molecular phylogeny of the genus *Anthurium*, based on chloroplast (*trnG* intron, *trnH–psbA* and *trnC–ycf6* intergenic spacers) and nuclear (first intron of *CHS* and partial flanking coding regions) DNA sequences (Carlsen 2011, Carlsen and Croat in press) shows that the palmately divided leaf morphology is homoplasious within the genus, having evolved at least three times independently, in Clades 3, 14 and 16 (Fig. 3).

Based on this molecular phylogeny (Carlsen 2011, Carlsen and Croat in press) (Fig. 3), some of the *Anthurium* species with palmately divided leaves previously recognized as section *Schizoplacium* (Schott 1860, Engler 1879, Engler 1905, Croat and Sheffer 1983), do not form a monophyletic group and are not even closely related to other palmately divided species. For example, *Anthurium pedatum*, a high elevation Colombian species with a highly divided palmatifid leaf blade, consistently clustered in the moderately supported Clade 14 (Fig. 3), along with *Anthurium furcatum* Sodiro, with trilobed leaves, and *Anthurium tremulum* Sodiro and *Anthurium macleanii* Schott, both with cordate leaves. Clade 14 is not easily characterized morphologically, although most of its species have hooded spathes and pendent spadices (Carlsen and Croat in press). Madison (1978) had pointed out the possible segregation of *Anthurium pedatum* from all other palmately divided *Anthurium* species by placing it alone in Group 5 of his revised classification. Molecular data now suggests that indeed *Anthurium pedatum* is not closely related to other palmately divided *Anthurium* species and therefore does not belong to section *Dactylophyllium* as currently defined here.

*Anthurium pedatoradiatum*, a Mexican species with palmatifid leaves and a member of section *Schizoplacium* (fide Schott 1860, Engler 1879, Engler 1905, Croat and Sheffer 1983), should also be removed from this group. Results of molecular analyses (Carlsen 2011, Carlsen and Croat in press) strongly suggest that it is more closely related to other northern Central American species (Clade 16) than to the clade of *Anthurium* species with palmately divided leaves (Clade 3) (Fig. 3). The strongly supported Clade 16, although quite variable in terms of leaf morphology, presents very uniform reproductive features, including only species that possess bright orange berries with a mealy mesocarp, characteristics also found in *Anthurium pedatoradiatum*. Madison (1978) previously separated *Anthurium pedatoradiatum* from the rest of palmately divided *Anthurium* species, and grouped it along with the other Mexican species with palmatifid leaves, *Anthurium podophyllum*, in his Group 4. The latter species have not been sampled for the current molecular phylogeny of *Anthurium* (Carlsen 2011, Carlsen and Croat in press). However, geographical affinities and similarities in fruit characteristics with other species of Clade 16 (Fig. 3) have made us consider that *Anthurium podophyllum* is also a member of this clade, and as such, it should be excluded from section *Dactylophyllium* as delimited here.

There are only four currently recognized species names included in the original description of Schott’s grex *Schizoplacium* (Schott 1860), all of which match well the protologue of the section. However, according to molecular studies (Carlsen 2011, Carlsen and Croat in press) (Fig. 3), *Anthurium pedatum*, *Anthurium pedatoradiatum*, and very likely *Anthurium podophyllum*, do not belong to the same clade and are not closely related to other palmately divided *Anthurium* species. Therefore, these three species are also excluded from section *Dactylophyllium* according to the circumscription presented here. Thus, of the initial group, only *Anthurium palmatum* remains. This climbing plant with elongated internodes and palmatifid leaves (Fig. 2C), restricted to the Lesser Antilles, is therefore here selected as the lectotype species for section *Schizoplacium*. Two other *Anthurium* species with palmatifid leaves (*Anthurium expansum* and *Anthurium longissimum*) (Fig. 1B) also belong to this section under its traditional circumscription (Table 1). *Anthurium palmatum* was not sampled in the current molecular phylogeny of the genus (Carlsen 2011, Carlsen and Croat in press) (Fig. 3), but the closely related *Anthurium longissimum*, with which it shares climbing habit, palmatifid leaf morphology, peduncle shorter than the petiole, green spathe, grayish purple spadix and reddish-purple berries, was used as a representative of this group of palmatifid species.

The molecular phylogeny of *Anthurium* (Carlsen 2011, Carlsen and Croat in press) clearly shows that most of the palmately divided species sampled in the study (except for *Anthurium pedatum* and *Anthurium pedatoradiatum*), belong in a single clade, Clade 3 (Fig. 3). These species were previously included in either section *Schizoplacium* (e.g. *Anthurium longissimum*, a representative of the group with palmatifid leaves) or section *Dactylophyllium* by Croat and Sheffer (1983). The findings of molecular analyses indicate that the group of species with palmatifid leaf morphology (i.e. *Anthurium longissimum*, *Anthurium palmatum* and *Anthurium expansum*) (Figs 1B, 2C), all sharing similar vegetative and reproductive characters, is not distinct from other species with palmately divided leaves. Thus, these two sections are here combined, and the morphological limits of this emended, more inclusive, group are redefined.

In terms of nomenclatural choice, since both names, *Schizoplacium* and *Dactylophyllium*, were published, albeit without a formal rank (i.e. as grex names), at the same time in Schott’s (1860) revision of the genus *Anthurium*, and were later simultaneously formalized as sections by Engler (1879), none of them has priority over the other. Therefore, in this study, section *Schizoplacium*, the smaller (probably containing only three currently accepted species names) and geographically more isolated group (mainly occurring in the Lesser Antilles and Cordillera de la Costa in Venezuela) has been placed into synonymy with the larger (probably including a total of 21 species, some undescribed) and more widespread group, section *Dactylophyllium*.

*Anthurium kunthii* (Fig. 2A) is here chosen as the lectotype for this emended section *Dactylophyllium* for several reasons. *Anthurium kunthii* was among the original species included in Schott’s (1860) first delimitation of the group and represents very well the morphological characters described in the protologue. Also, this species was sampled in the current molecular phylogeny of the genus (Carlsen 2011, Carlsen and Croat in press) (Fig. 3), and it clearly belongs to the group of species with palmately divided leaves in Clade 3. Additionally, *Anthurium kunthii* is among the oldest species described within the group (in 1845) (Table 1), but unlike *Anthurium digitatum* (the oldest described species, from 1829), its taxonomic status as a species has not been previously questioned.

The following section provides an updated description of *Anthurium* section *Dactylophyllium* (Schott) Engler, emend. Croat & Carlsen, and lists all currently recognized species now comprising this group and their known geographic distribution.

## Taxonomic treatment

### Anthurium Dactylophyllium

(Schott) Engler, emend. Croat & Carlsen, Prodr. Syst. Aroid. 542. 1860. Lectotype (designated here): Anthurium kunthii Poepp., Nov. Gen. Sp. Pl. 3: 84–85. 1845.

Figures 1 , 2

Anthurium grex *Schizoplacium* Schott, Prodr. Syst. Aroid. 538. 1860. Lectotype (designated here): *Anthurium palmatum* (L.) Schott, Wiener Z. Kunst 1829(3): 828. 1829.

#### Remarks.

Mostly appressed-climbing or scandent plants with internodes usually longer than broad, or terrestrial short stemmed plants; roots moderately sparse at each node on climbing plants, sometimes moderately dense on terrestrial species with short internodes; cataphylls usually persisting as fibers, sometimes deciduous, rarely persisting intact, the cataphyll fibers typically pale, sometimes dark reddish brown; petioles typically subterete, usually at least weakly sulcate adaxially, typically drying greenish to gray-green, sometimes dark brown; blades palmately divided and deeply lobed with 5–7 lobes united at the base (i.e. palmatifid leaves) (Figs 1B, 2C) (*Anthurium expansum*, *Anthurium longissimum*, and *Anthurium palmatum*) or palmatisect with segments (leaflets) divided completely to base and free (Fig. 2A–B), sometimes 3-sect (Fig. 1C) (*Anthurium arisaemoides* Madison, *Anthurium thrinax* Madison, *Anthurium triphyllum*, and *Anthurium trisectum* Sodiro), more commonly 5–11-sect (Fig. 1A, D) (*Anthurium brevipedunculatum* Madison, *Anthurium clavigerum*, *Anthurium croatii* Madison, *Anthurium eminens*, *Anthurium kunthii*, *Anthurium pentaphyllum*, *Anthurium polyschistum* R.E. Schultes & Idrobo, and *Anthurium sinuatum*), the petiolules of each segment short or long (Fig. 2A–B), the segments usually entire, sometimes sinuate (*Anthurium clavigerum*, *Anthurium sinuatum*) or weakly to strongly pinnately lobed (*Anthurium clavigerum*); the medial segment or lobe largest; side segments or lobes diminishing in size; juvenile blades simple; leaf surface usually smooth, glabrous, generally drying greenish, sometimes yellow-brown or dark brown; midrib typically raised on both surfaces; primary lateral veins typically conspicuous, usually well spaced, weakly raised or sunken above, usually narrowly rounded and prominently raised below; tertiary veins typically visible, sometimes moderately well-raised beneath. INFLORESCENCE short- (*Anthurium brevipedunculatum*, *Anthurium pentaphyllum*) or more commonly long-pedunculate; spathe typically green, spreading, sometimes ovate and erect (*Anthurium brevipedunculatum*), usually persistent; spadix green to purplish violet, usually long-tapered, sometimes short-tapered. FRUITS purple, violet-purple or reddish-purple berries.

Species of *Anthurium* included in section *Dactylophyllium*, under this revised delimitation, are mainly distributed in the Amazon lowlands, with a few widespread species ranging into Central America (*Anthurium clavigerum*, *Anthurium kunthii*, and *Anthurium trisectum*), and into the Atlantic coast of South America to Brazil (*Anthurium pentaphyllum*). Three taxa have disjunct distributions in the coastal mountain ranges of the Cordillera Central of Venezuela (*Anthurium digitatum* and *Anthurium longissimum*) and the Lesser Antilles (*Anthurium palmatum*).

Presently, 24 accepted taxa (20 species and 4 varieties or subspecies) occur in section *Dactylophyllium* as emended here. These taxa and their geographic distribution are as follow:

*Anthurium arisaemoides* Madison (Ecuador, Peru)

*Anthurium brevipedunculatum* Madison (Bolivia, Brazil, Colombia, Ecuador, Peru)

*Anthurium buchtienii* K.Krause (Bolivia)

*Anthurium clavigerum* Poepp. (widespread, Nicaragua to Venezuela and Peru)

*Anthurium croatii* Madison (Bolivia, Brazil, Colombia, Ecuador, Peru)

*Anthurium digitatum* (Jacq.) Schott (Venezuela)

*Anthurium eminens* Schott var. *eminens* (Bolivia, Brazil, Colombia, Ecuador, French Guiana)

*Anthurium eminens* Schott var. *longispadix* Croat & M.Mora (Colombia)

*Anthurium expansum* Gleason (French Guiana, Guyana, Suriname, Venezuela)

*Anthurium kunthii* var. *cylindricum* Croat (Bolivia)

*Anthurium kunthii* Poepp. var. *kunthii* (Costa Rica to Peru and Bolivia) (Fig. 2A)

*Anthurium longissimum* Pittier ssp. *longissimum* (Venezuela) (Fig. 1B)

*Anthurium longissimum* Pittier ssp. *nirguense* Bunting (Venezuela)

*Anthurium moonenii* Croat & E.G.Gonçalves (French Guiana)

*Anthurium palmatum* (L.) Schott (Lesser Antilles) (Fig. 2C)

*Anthurium pentaphyllum* (Aubl.) G. Don var. *bombacifolium* (Schott) Madison (Belize, Costa Rica, Guatemala)

*Anthurium pentaphyllum* (Aubl.) G.Don var. *pentaphyllum* (widespread Costa Rica to the Guianas, Brazil and Peru) (Figs 1A, 2B)

*Anthurium polydactylum* Madison (Bolivia, Peru) (Fig. 1D)

*Anthurium polyschistum* R.E. Schultes & Idrobo (Brazil, Colombia, Ecuador, Peru)

*Anthurium sinuatum* Benth ex Schott (Brazil, French Guiana, Suriname, Venezuela)

*Anthurium thrinax* Madison (French Guiana, Guyana)

*Anthurium triphyllum* (Willd. ex Schult.) Brongn. ex Schott (Bolivia, Ecuador, Peru)

*Anthurium trisectum* Sodiro (Costa Rica to Ecuador) (Fig. 1C)

*Anthurium zuloagae* Croat (Colombia)

There are also at least four more currently undescribed species in the section, and at least two more varieties that need formal recognition. A complete taxonomic revision, including identification keys, species synonymy, descriptions and illustrative photographs, of all the species of *Anthurium* with palmately divided leaves comprising the newly amended section *Dactylophyllium* is indeed needed, but beyond the scope of this article.

## Supplementary Material

XML Treatment for Anthurium Dactylophyllium
